# Effect of a proprietary *Magnolia *and *Phellodendron *extract on stress levels in healthy women: a pilot, double-blind, placebo-controlled clinical trial

**DOI:** 10.1186/1475-2891-7-11

**Published:** 2008-04-21

**Authors:** Douglas S Kalman, Samantha Feldman, Robert Feldman, Howard I Schwartz, Diane R Krieger, Robert Garrison

**Affiliations:** 1 Director, Nutrition, Miami Research Associates. Miami, FL, USA; 2 Senior Coordinator, Nutrition, Miami Research Associates. Miami FL, USA; 3 Director, Women's Health, Miami Research Associates. Miami, FL, USA; 4 Medical Director, Miami Research Associates. Miami, FL, USA; 5 Medical Director, Nutrition & Endocrinology, Miami Research Associates. Miami, FL, USA; 6 Chairman of the Board (deceased), Next Pharmaceuticals, Salinas, CA, USA

## Abstract

**Background:**

Recent research has established correlations between stress, anxiety, insomnia and excess body weight and these correlations have significant implications for health. This study measured the effects of a proprietary blend of extracts of *Magnolia officinalis *and *Phellodendron amurense (*Relora^®^) on anxiety, stress and sleep in healthy premenopausal women.

**Methods:**

This randomized, parallel, placebo controlled clinical stud**y **was conducted with healthy, overweight (BMI 25 to 34.9), premenopausal female adults, between the ages of 20 and 50 years, who typically eat more in response to stressful situations and scores above the national mean for women on self-reporting anxiety. The intervention w**as **Relora (250 mg capsules) or identical placebo 3 times daily for 6 weeks. Anxiety as measured by the Spielberger STATE-TRAIT questionnaires, salivary amylase and cortisol levels, Likert Scales/Visual Analog Scores for sleep quality and latency, appetite, and clinical markers of safety. The study was conducted by Miami Research Associates, a clinical research organization in Miami, FL.

**Results:**

The intent-to-treat population consisted of 40 subjects with 26 participants completing the study. There were no significant adverse events. Relora was effective, in comparison to placebo, in reducing temporary, transitory anxiety as measured by the Spielberger STATE anxiety questionnaire. It was not effective in reducing long-standing feelings of anxiety or depression as measured using the Spielberger TRAIT questionnaire. Other assessments conducted in this study including salivary cortisol and amylase levels, appetite, body morphology and sleep quality/latency were not significantly changed by Relora in comparison to placebo.

**Conclusion:**

This pilot study indicates that Relora may offer some relief for premenopausal women experiencing mild transitory anxiety. There were no safety concerns or significant adverse events observed in this study.

## Background

Recent research has established correlations between stress, anxiety, insomnia and excess body weight and these correlations have significant implications for health. Substantial and prolonged stress and anxiety can cause adverse health effects to the immune function, hormone levels, enzymes and gastrointestinal function. Recently links have been established between chronic stress and obesity [1,2]. Increased levels of the stress hormone, cortisol, have been correlated to increased eating of high caloric foods and sweets. Additionally, among people who identified themselves as "stress eaters," weight gain tended to increase over time [3]. It is also known that stress worsens insomnia, and insomnia has been linked to overweight and obesity [4,5]. Among 6,115 people, ages 32–59, comparisons were made between the weights of those who slept for a normal period of time (7–9 hours) and those who slept for shorter or longer periods of time. Those who slept for shorter periods of time than normal were more likely to be obese and those who slept for longer periods of time were less likely to be obese. Those who slept 2–4 hours per night, 5 hours per night or 6 hours per night were respectively 73%, 50% and 23% more likely to be obese. Those who slept for 10 or more hours were 11% less likely to be obese [5].

Interventions for stress and anxiety range from nutritional support to the use of medications such as benzodiazepines and selective serotonin reuptake inhibitors. Recently a United States Patent (No 6,582,735) was granted for the use of an extract of *Magnolia officinalis *bark for stress related conditions involving elevated cortisol, such as control of body weight, sleep disturbances and restlessness.

Extracts of *Magnolia officinalis *bark and its active constituent, honokiol, have been studied in various mouse models that have compared the activity to diazepam (a benzodiazepine anxiolytic used to treat anxiety disorders since the 1960's) [6-8]. The studies found that honokiol had an anxiolytic effect without the common side effects of diazepam (motor dysfunction, sedation or amnesia). The *Magnolia officinalis *bark extract and an extract of *Phellodendron amurense *bark were tested in an animal model for stress, the Chick Social Separation Stress Procedure, with positive results [9]. Both extracts reduced distress vocalization and stress-induced analgesia without causing sedation. Berberine, a constituent of the *Phellodendron *extract, has demonstrated a significant anxiolytic effect in the black and white and the elevated plus maze tests in mice [10]. Berberine has also demonstrated an antidepressant effect in the forced swim test in mice [11].

The subject of this study, Relora^® ^(Next Pharmaceuticals, Inc, Salinas, CA), is a proprietary dietary supplement formulation consisting of a blend of extracts of *Magnolia officinalis *bark and *Phellodendron amurense *bark standardized to honokiol and berberine, respectively.

In previously conducted trials by the study sponsor (unpublished in-house data), Relora has demonstrated some efficacy for reducing perceived anxiety and enhancing feelings of well-being. One study also measured the effects of Relora on salivary cortisol and found that it had some effect on morning cortisol while also raising dehydroandrostenedione levels (DHEA).

The purpose of this study was to conduct a placebo-controlled clinical trial to determine the effects of Relora on anxiety using psychometric questionnaires, cortisol levels and sleep (questionnaires). A part of this study that investigated the effects of Relora on stress-related changes in body weight has previously been published [12].

## Materials and methods

### Test Material

Relora^® ^(NP 33–39) is a proprietary blend of a patented extract of the bark of *Magnolia officinalis *Rehder & Wilson [Magnoliaceae] (US Patent Nos. 6,582,735 and 6,814,987) and an extract of the bark of *Phellodendron amurense *Rupr. [Rutaceae]. The product is standardized to not less than 1.5% honokiol and 0.1% berberine. Subjects ingested 250 mg in dark red opaque capsules three times daily or a placebo that was identical in size, shape and color.

### Study Design

In a prospective, two-group, parallel, randomized, double-blind, placebo-controlled trial design, healthy adult females with anxiety who reported that they typically eat more in response to stressful situations were enrolled and followed for six weeks. Participants were split into two groups of 20 using block code randomization (coded labels provided by the manufacturer) and then administered either Relora or a matched placebo for six weeks. The study was approved by the Integreview Ethical Review Board, Austin, TX.

Recruitment advertisements were placed in English and Spanish in local newspapers in the Miami, FL, area. The advertisements read, "Is stress making you eat? You may qualify to participate in a research study of a nutritional supplement for people who eat more during stressful situations. To qualify you must not be taking medications for depression or anxiety, be overweight but in good general health, be 20 to 50 years of age, and be a premenopausal female." The subjects for the study were pre-screened by phone and potential candidates were called in for a screening and baseline evaluation (Visit 1) after providing written informed consent. Acceptable subjects were enrolled (Visit 2), and then randomized with equal probability to receive either Relora or placebo. Efficacy and safety evaluations were performed during visits at treatment weeks 3 and 6 (Visits 3 and 4).

To be included in the study, subjects had to be healthy overweight females (BMI 25 – 39.9 kg/m^2^), be premenopausal (aged 25 to 50) and to self report increased eating in response to stress. Subjects were excluded if they had personal history of heart disease, uncontrolled high blood pressure, renal or hepatic impairment/disease, Type I or II diabetes, psychiatric disorders, cancer (except basal cell carcinoma of > 5 years ago), use of any monoamine oxidase inhibitor medication, sleep disorders, glaucoma, gastric ulcer or reflux disease or a seizure disorder, unstable thyroid disease, pregnancy, lactation, or any medical condition deemed exclusionary by the medical staff. Scores positive for Binge Eating Disorder utilizing the Diagnostic and Statistical Manual of Mental Disorders (DSM-IV) criteria for diagnosis (score > 7), or scores positive for clinical depression via the Center for Epidemiologic Studies Depression Scale (CES-D) questionnaire (scores >16) or score below the norm for women 30 – 39 years of age on the State-Trait Anxiety Inventory (STAI) (< 34; per investigator discretion) or if the subject scored positively for severe anxiety they were also excluded from this study. Psychiatric disorders were determined by medical history, rechecked by the Principal Investigator (a medical doctor), and by means of questionnaires given by trained professionals. Subjects were also excluded if they were on monoamine oxidase inhibitors (MAO-I), anxiolytics, psychotropics including SSRI's, daily use of OTC medications or were currently taking stimulant medications (prescription, self-prescribed or OTC). In addition, recent use of weight control agents, unstable body weight, use of any form of a steroid medication, use of any type of dietary supplement were also excluded. Women who were pregnant, lactating, planning to become pregnant during the study or not using an acceptable form of contraceptive device or if a subject was ever diagnosed with Post-traumatic stress disorder (PTSD), they were excluded from this study. Study subjects were not allowed to take supplements that are, or were purported, to affect mood states. They were also not allowed to start any new supplements. If a subject was already taking a multivitamin/mineral supplement, they were allowed to continue doing so. To ensure compliance, a dichotomous questionnaire was used by the coordinator at each visit.

### Analyses

Blood was drawn for analysis of biochemical markers for safety parameters at the beginning and end of the study. A 3-day (2 working days and 1 non-working day) food diary was recorded by the subjects at baseline and post-treatment. At the beginning, mid-point and end of the study, participants were weighed and tested using psychometric questionnaires. The psychometric questionnaires used in this study included: Spielberger STATE-TRAIT (20-item questionnaires by which subjects describe transient or general anxiety, on a 4-level scale); Visual Analog Scale – Sleep Quality (VAS-SQ; 10 cm scale, self-reported 5 point Likert scale, 0 = no sleep, 4 = very restful sleep); Sleep Latency Questionnaire – asked each time the VAS-SQ was conducted; subjective notion of how long, on average it takes the person to fall asleep, in minutes); and subjects were also given 3-day saliva collection kits (to be analyzed for salivary amylase and salivary cortisol) at the screening and mid-study visits (with samples taken at three different times (upon waking, 30 minutes later, and in the evening) for three consecutive days during each collection cycle), to be collected at the randomization and end-of-study visits, respectively.

Participants were also asked to follow their typical diets and exercise routines. Physical exercise quantity was quantified with the Framingham Physical Activity Index.

### Statistical Plan

The primary efficacy analyses were conducted on an Intent-to-Treat basis, with missing observations imputed by the "last observation carried forward" (LOCF) method. Safety analyses were conducted on all subjects who received study product. Unpaired *t*-tests were used to compare changes over time between the two groups, except for variables that were substantially non-normally distributed, in which case the Mann-Whitney U-Test was employed. Statistical analysis was conduced using Excel 2002, SPSS version 13.0 and "R" version 2.1.1.

The number of participants for this study was pre-determined as a convenience sample. It was noted that a sample size of 20 subjects in each of two groups could provide 80% probability to produce a significant result (P < 0.05) in an unpaired comparison, when the mean between-group difference in any endpoint is 0.9 as large as the within-group standard deviation for that endpoint.

## Results

### Study Population

Forty two female subjects, aged 25–50 (mean 38.6 ± 6.1, BMI of 31.2 ± 4.1 kg/m^2^) were enrolled and randomized into two test groups. Two of the subjects were subsequently discovered to be outside of the inclusion/exclusion criteria and were subsequently excluded from all but safety analysis. There were no statistical differences between groups at baseline in scores for age, ethnicity, anthropometry (height, weight, BMI, waist and hip circumference and waist-to-hip ratio). There were no clinical differences in vital signs (heart rate, blood pressure) or lab tests (glucose, creatinine, blood urea nitrogen, alkaline phosphatase, aspartate aminotransferase (AST), alanine aminotransferase (ALT), white blood cell count, hemoglobin, hematocrit, thyroid stimulating hormone). There was one slight statistical difference in serum ALT (17.4 ± 8.4 vs 23.6 ± 10.1 IU, respectively for treatment and placebo; p = 0.032) but this was not considered of clinical importance. There were no statistical differences between the two groups in the psychometric measures listed in the Methods section.

The intent-to-treat population consisted of 40 subjects. Sixteen participants in the treatment group and ten participants in the control group completed the study. Nine subjects, one in the treatment group and eight in the control group, were lost to follow-up. Five subjects, three in the treatment group and two in the placebo group, terminated early for various reasons. Three subjects withdrew due to adverse events: two in the treatment group, one in the placebo group (see Table 1). The researchers could not determine the reason for discrepancy in the difference in dropouts between placebo and the active groups. Compliance in the intent-to-treat population was 90.6 ± 11.5% in the Relora group and 86.1 ± 23.4% in the placebo group, with no statistical difference between them.

**Table 1:** Disposition of Enrolled Subjects

**Disposition**	**Relora**	**Placebo**	**Total**	**p value**
Completed per protocol	16 (80%)	10 (50%)	26 (65%)	
Early Termination	3 (15%)	2 (10%)	5 (12%)	**0.030**
Lost to Follow-up	1 (5%)	8 (40%)	9 (22%)	
Total	20 (100%)	20 (100%)	40 (100%)	

Disposition of enrolled subjects is listed in the table with the percentage of the total listed in parenthesis. The p-value in the column on the right compares those who terminated early, based upon the Fisher Exact test.

### Safety

The safety population consisted of all subjects who consumed any product and who had some post-dose follow-up safety observations. It included 39 subjects. No serious adverse events were noted in this study. Two subjects in the treatment group and one in the placebo group dropped out of the study early due to adverse events. One subject in the Relora group complained of heartburn, hands shaking, perilabial numbness, sexual dysfunction, and thyroid dysfunction. The second subject in the Relora group complained of fatigue and headache. The subject in the placebo group complained of irritability, abdominal bloating and tiredness. No significant changes were noted in vital signs during the course of the study. No laboratory values showed significant differences between treatment and placebo in mean changes from baseline to the end of the study.

### Efficacy

The primary objective of the study was to determine the effects of Relora, compared to placebo, on mild anxiety. The Spielberger STATE and TRAIT anxiety questionnaires were given to subjects at screening and after 6 weeks of treatment. There were significant decreases (less anxiety) in the total STATE score over time for both treatment and placebo groups with the average decrease being almost twice as much for the Relora group (-14.3 ± 12.1 and -7.6 ± 9.8, respectively). The reduction in anxiety was significantly greater for the treatment group compared to the placebo group (p = 0.045) (Table 2).

**Table 2:** Spielberger State Total Score

**Time Point**	**Relora (N = 20)**	**Placebo (N = 20)**	**Comparison p values**
Screening	32.5 ± 7.6 33 (19 – 47)	29.4 ± 6.7 29.5 (18 – 43)	0.166
Week 6	18.2 ± 8.6 17.5 (1 – 38)	21.7 ± 9.8 20.5 (1 – 43)	0.245
Change from Baseline to Week 6	-14.3 ± 12.1 -16 (-34 – 9) **p < 0.001**	-7.6 ± 9.8 -2.5 (-34 – 2) **p = 0.005**	**0.045**

Values are listed in the following format: Mean ± Standard Deviation; Median (Minimum-Maximum). The values in the bottom row are average changes from baseline and the p-values are based upon single-group Student *t*-tests or non-parametric equivalent. P-values listed in the column to the right compare the two groups and are based upon an unpaired Student *t*-test or non-parametric equivalent.

According to the Spielberger TRAIT questionnaire, Relora significantly reduced general and longer lasting anxiety over the 6 weeks (-12.2 ± 10.4; p < 0.001). The placebo group also had a significant decrease in score (-7.6 ± 10.2; p = 0.004). When the average change over time for the two groups was compared, there was no significant difference between the two (Table 3).

**Table 3:** Spielberger Trait Total Score

**Time Point**	**Relora (N = 20)**	**Placebo (N = 20)**	**Comparison p values**
Screening	38.5 ± 10.6 41.5 (20 – 59)	36.5 ± 9.9 36.5 (12 – 53)	0.531
Week 6	26.4 ± 5.6 25.5 (19 – 42)	28.9 ± 10.1 26 (12 – 53)	0.341
Change from Baseline to Week 6	-12.2 ± 10.4 -12 (-31 – 7) **p < 0.001**	-7.6 ± 10.2 -2.5 (-35 – 0) **p = 0.004**	0.156

Values are listed in the following format: Mean ± Standard Deviation; Median (Minimum-Maximum). The values in the bottom row are average changes from baseline and the p-values are based upon single-group Student *t*-tests or non-parametric equivalent. P-values listed in the column to the right compare the two groups and are based upon an unpaired Student *t*-test or non-parametric equivalent.

At the end of the study, the subjects were given a questionnaire to assess their perceived stress or anxiety. The Relora group reported that they experienced a significant reduction in perceived anxiety/stress (p = 0.043) compared to placebo (see Table 4). The subjects were asked: On a scale of 1 to 5 with 1 being the lowest possible difference and 5 being the best change or difference: Do you feel that taking the supplement helped to reduce your perceived stress or anxiety? The Relora group average score was a 4, while placebo average score was a 2.

**Table 4:** Subjective End-of-Study Assessment on Perceived Stress/Anxiety

**Relora (N = 19)**	**Placebo (N = 11)**	**p value**
3.84 ± 1.38 4 (1 – 5)	2.64 ± 1.69 2 (1 – 5)	**0.043**

Values are listed in the following format: Mean ± Standard Deviation; Median (Minimum-Maximum). The p-value listed in the column to the right compares the two groups and is based upon an unpaired Student *t-*test or non-parametric equivalent.

Levels of salivary cortisol and amylase were measured. Samples were taken at three different times of the day (upon waking, 30 minutes later and in the evening) for three consecutive days during two collection cycles (baseline and at six weeks). There was a trend towards decreases in cortisol levels at all time points in both groups, but none of these changes reached statistical difference and there was no significant difference between groups. Salivary amylase levels increased significantly in the evening in the treatment group (change from 29.8 ± 23.5 to 37.8 ± 31.5, a total change of 8.0 ± 12.4, p = 0.025), but this change was not significant when compared to the placebo group.

Appetite levels, dietary restraint, as well as intakes of calories, protein, carbohydrate and fat were measured at the beginning and the end of the study but there were no consistent changes when the Relora and placebo groups were compared. There were also no statistical changes in weight, waist circumference, hip circumference or waist/hip ratio when the two groups were compared.

Quality of sleep did not change statistically for either group and there was no difference between groups. Sleep latency was determined by a questionnaire: During the past month, how long (in minutes) has it usually taken you to fall asleep each night? Subjects in the treatment group reported a decrease in sleep latency (change from 33.7.8 ± 29.5 to 22.6 ± 22.1, a total decrease of 11.1 ± 17.2 minutes, p = 0.012), but this change was not significant when compared to the placebo group.

## Discussion

Relora^® ^significantly reduced anxiety compared to placebo as determined by the Speilberger STATE anxiety questionnaire. This questionnaire measures temporary, transitory anxiety that includes feelings of apprehension, tension, nervousness and worry that increase in response to physical danger and psychological stress. The questionnaire measures how the respondents feel "right now, at this moment" [13]. This measurement has been used in counseling and to measure the effects of relaxation training.

General, consistent, long-standing feelings of anxiety or depression were measured using the Spielberger TRAIT questionnaire. In this questionnaire, psychoneurotic and depressed patients tend to have high scores [13]. According to this measurement, Relora and placebo each significantly reduced general anxiety over the 6 weeks. There was no statistical difference compared to the placebo group.

The State-Trait Anxiety Inventory (STAI) was initially conceptualized as a research instrument for the study of anxiety in adults. It is a self-report assessment device which includes separate measures of state and trait anxiety. Scores on the STAI have a direct interpretation: high scores on their respective scales mean more trait or state anxiety and low scores mean less. Both percentile ranks and standard scores are available for male and female working adults in three age groups (19–39, 40–49, 50–69). The stability of the STAI scales have been assessed on male and female samples of high school and college students for test-retest intervals ranging from one hour to 104 days. The magnitude of the reliability coefficients decreased as a function of interval length. For the Trait-anxiety scale the coefficients ranged from 0.65 to 0.86, whereas the range for the State-anxiety scale was 0.16 to 0.62. This low level of stability for the State-anxiety scale is expected since responses to the items on this scale are thought to reflect the influence of whatever transient situational factors exist at the time of testing. Correlations between this scale and other measures of trait-anxiety: the Taylor Manifest Anxiety Scale, the IPAT Anxiety Scale, and the Multiple Affect Adjective Check List are 0.80, 0.75, and 0.52, respectively [11].

Thus, in this short 6-week study Relora was effective, in comparison to placebo, in reducing temporary, transitory anxiety as measured by the Spielberger STATE anxiety questionnaire. It was not effective in reducing long-standing feelings of anxiety or depression as measured using the Spielberger TRAIT questionnaire. Relora was also effective in reducing self-perceived stress or anxiety. These results are consistent with those observed in the uncontrolled open label studies previously conducted.

Other assessments conducted in this study including salivary cortisol and amylase levels, appetite, body morphology and sleep quality/latency were not significantly changed by Relora in comparison to placebo. There are trends that suggest that a larger study conducted over a longer period of time might show efficacy in these variables, as well.

## Conclusion

This pilot study indicates that Relora^® ^may offer some relief for premenopausal women experiencing mild transitory anxiety. There were no safety concerns or significant adverse events observed in this study. Further studies with a larger number of participants are recommended to explore the anxiolytic effect of Relora and to determine whether that effect may be accompanied by a reduction in stress, in appetite, and an improvement in sleep.

## Competing interests

This research was supported by a grant from Next Pharmaceuticals, Salinas, CA. Statistical consultation and manuscript preparation was also funded by Next Pharmaceuticals.

## Authors' contributions

DSK, DRK and RG contributed to the design of the study, SF contributed to the coordination of the study, and DSK and SF collected study data. DRK was the principal investigator, DSK, RF and HS were sub-investigators.
